# Features of omental adipose tissue in endometrial cancer patients with ‘standard’ or ‘metabolically healthy’ obesity: associations with tumor process characteristics

**DOI:** 10.1186/s40064-016-3582-6

**Published:** 2016-10-31

**Authors:** Lev M. Berstein, Aglaya G. Iyevleva, Marina S. Mukhina, Dmitry A. Vasilyev, Tatyana E. Poroshina

**Affiliations:** 1 Laboratory Oncoendocrinology, Pesochny, N.N.Petrov Research Institute of Oncology, Leningradskaya 68, St.Petersburg, 197758 Russia; 2 All-Russian Scientific Centre of Radiology and Surgical Technologies, St.Petersburg, Russia

**Keywords:** Endometrial cancer, Obesity phenotype, Omental fat, UCP1, adipokines, Macrophages, Inflammation

## Abstract

**Purpose:**

Adipose tissue products may contribute to endometrial cancer (EC) initiation and further growth that encourages the analysis of this issue in patients with different obesity phenotypes.

**Methods/patients:**

Omental fat depot characteristics were studied in EC patients (n = 57) with “standard” (SO) or “metabolically healthy” (MHO) obesity. Collected omental samples were evaluated by immunohistochemistry /IHC/ for brown fat marker UCP1, CYP19 (aromatase) and macrophage infiltration markers (CD68, CD163, crown-like structures/CLS) expression. Total RNA extracted from the same samples was investigated for UCP1, CYP19, PTEN and adipokine omentin mRNA.

**Results:**

Immunohistochemistry data revealed a statistically significant increase in aromatase and CD68 expression and tendency to increase of UCP1 expression in SO patients’ omental fat compared to samples obtained from MHO patients. Additionally, positive correlation of EC clinical stage with UCP1 protein and its mRNA content in omental fat was pronounced in MHO as well as SO group, while with omentin mRNA it was discovered only in patients with SO. An inclination to the correlation with better tumor differentiation was seen for UCP1 and CD68 protein expression in patients with MHO and with worse (high grade) differentiation—for CD68 expression in the group with SO.

**Conclusions:**

In aggregate, this suggests that obesity phenotype has significant impact on omental fat tissue characteristics which is related to the clinical course of EC and may have practical consequences.

## Background

Endometrial cancer (EC) is the most common gynecological cancer. Therefore, it has now long been a point of interest not only of oncologists and gynecologists, but also of specialists in other fields. One of most popular concepts of EC biology concerns its connections with obesity to which—especially in the last years—even a causal role has been rather often imputed [see (Byers and Sedjo 2015)]. Concerning an importance of this aspect, we should mention current tendencies, according to which by 2030 a roughly identical and noticeable (about 50–60%) increase is expected in primary EC cases number (Sheikh et al. 2014) as well as in obesity prevalence (Kelly et al. 2008). Herewith, although for several decades a concept of obesity being primarily the predisposing factor mainly for EC of type I dominated (Bokhman 1983; Sherman 2000; Brinton et al. 2013), there are presently some new publications advocating no significant difference between type I and type II EC in this context (Setiawan et al. 2013).

Besides being the possible consequence of gradual changes in EC biology (Evans et al. 2011; Berstein et al. 2015), the latter ascertaining may also be related to another aspects of obesity problem and its study in uterine body cancer patients. In particular, we must to stress that obesity and high body mass index are not equivalent entities (Crosbie et al. 2012) and that obesity draws attention not only due to the growing scale of its epidemics (Kelly et al. 2008), but also by the fact of its *heterogeneity* (Berstein 2012). Due to this, obesity is not always univocal in its manifestations and consequences, justifying in the first place the distinguishing between “standard” (SO), or insulin resistance-associated, and “metabolically healthy” (MHO) obesity phenotypes (Sims 2001; Karelis 2008) and underlining the need in their comparative considering in cancer patients including patients with EC (Berstein et al. 2015; Berstein 2012; Calori et al. 2011).

It should be added that for determining of obesity role as an EC risk factor and its clinical course modifier (Brinton et al. 2013; Setiawan et al. 2013; Fader et al. 2009) an undeniable significance belongs to adipose tissue studies, where particular attention may attract a greater omentum fat depot located rather close to the uterus. Among other things, this corollary may be derived from uncoupling protein 1 (UCP1) research. While this mitochondrial protein is involved in thermogenesis and energy expenditure, finding of its mRNA in omental fat (Oberkofler et al. 1997) leads to conclusion that the latter can possess the properties of both, white and brown adipose tissue. Although functional roles of these tissues are supposed to be directly opposite in view of obesity prevention problem (Cypess and Kahn 2010), this concept is not yet conventional (Jensen 2015) and needs further elaboration [that is also true in regard of brown fat associations with cancer (Berstein 2012)]. On the other hand, the greater omentum fat depot belongs formally to visceral fat, which is supposed to be linked with insulin resistance and is capable to produce a set of adipokines (Ibrahim 2010), although the characteristics of these adipokines vary significantly and some of them, in particular omentin, are mostly produced by stromal cells of adipose tissue (Yang et al. 2006).

In accordance with said above, our research was aimed at studying omental fat characteristics (in particular, expression of UCP1, omentin, aromatase/estrogen synthetase, certain macrophage infiltration markers, etc.) in EC patients with “standard” (SO) and “metabolically healthy” (MHO) obesity and at evaluation of these characteristics relation to cancer process features.

## Methods and patients

A total of 57 treatment-naïve patients (mean age of 60.1 years) mostly with early EC stages (43 with Ia-c, 9 with IIa-b, 5 with IIIa-b) were enrolled. About two-thirds of tumors were endometrioid adenocarcinomas. Besides EC stage and morphology, its differentiation grade (G-grade) was determined. In 2–3 days before surgical intervention anthropometric values (body mass, height, body mass index/BMI/) were evaluated, and body fat content was determined by bioelectrical impedance test. At the same time point hormone-metabolic status of patients was assessed. It included evaluation of fasting serum glucose, cholesterol and triglycerides values (Vector-Best kits, Novosibisk, Russia), as well as insulin (by ELISA kit of DRG, Germany) and omentin (ELISA kit of BioVendor, Chech Republic) serum values. The insulin resistance index (HOMA-IR) value was calculated according to the formula: fasting insulin (μU/l) × fasting glucose (nmol/l)/22.5 (Matthews et al. 1985).

Patients were divided into groups according to BMI values: <25.0, 25.0–29.9 and ≥30.0. Among patients with BMI values of ≥25.0 the ones with clinical signs of MHO or SO were detected. The first group consisted of EC patients not having at least three of the following five criteria: impaired glucose tolerance/hyperinsulinemia, hypertriglyceridemia, waist circumference increase, low serum high-density cholesterol values and arterial hypertension. These criteria were chosen in accordance with current guidelines (Samocha-Bonet et al. 2014).

**Fig. 1:**
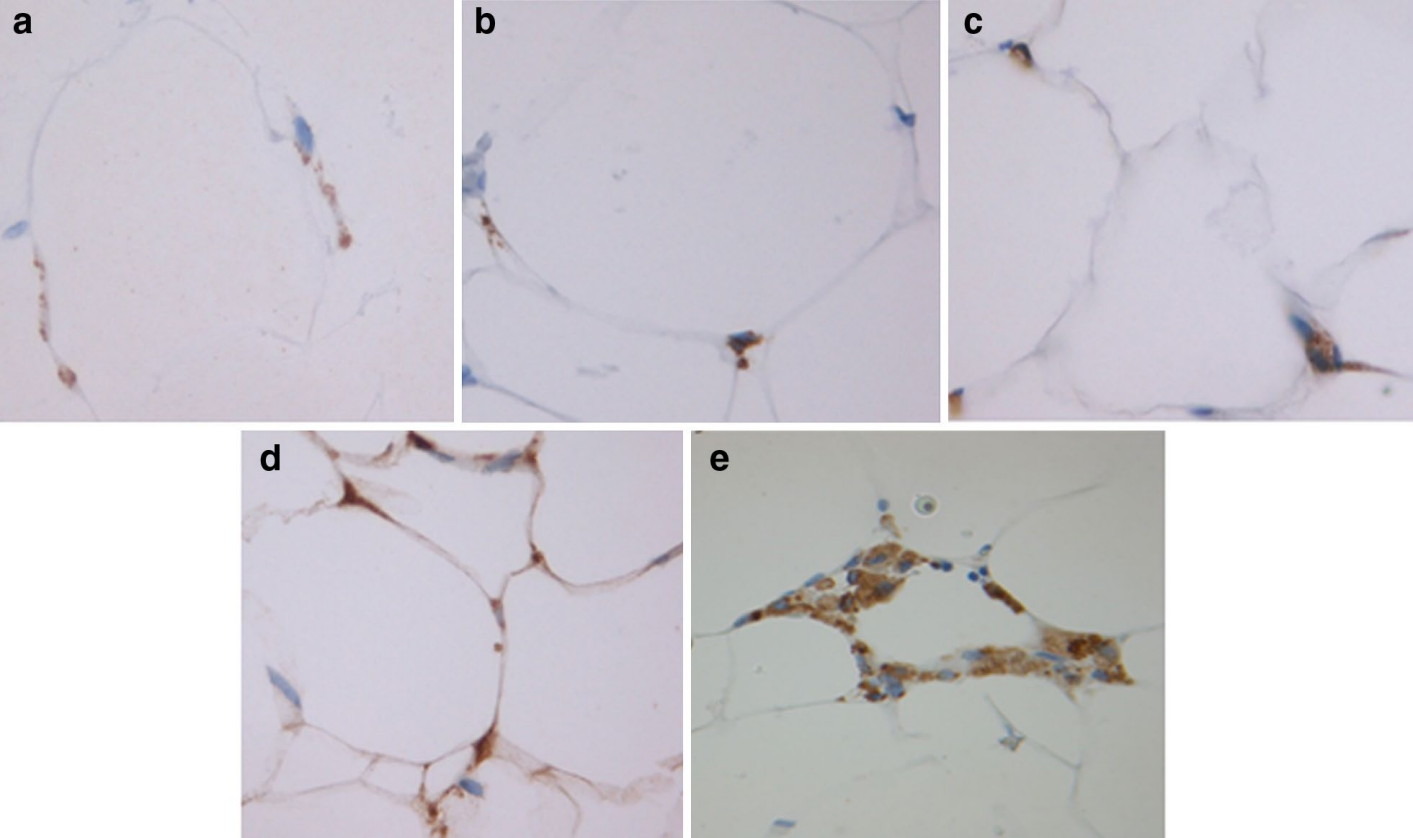
Examples of IHC staining of omental fat in endometrial cancer patients **a** CD68 **b** CD163 **c** UCP1 **d** Aromatase **e** Corona-like structures (CLS). Magnification ×400

Omental fat samples (not having signs of metastases and taken during surgical intervention approximately from the same inferior area of big omentum in practically all patients) were fixed in 10% formalin for further paraffin embedding and immunohistochemistry assay (n = 50). Analogous samples (n = 57) were instantly placed in liquid nitrogen and stored at −70 °C for further total RNA extraction. The immunohistochemical expression of mitochondrial UCP1 protein (polyclonal rabbit antibodies, Thermo Scientific; dilution 1:500), as well as aromatase (estrogen synthetase) (polyclonal rabbit antibodies Abcam 65693, 1:50), macrophage markers CD68 (polyclonal rabbit antibodies, Dako, 1:200) and CD163 (monoclonal mouse antibodies, Novocastra, 1:100) was studied in deparaffinized sections of omental fat tissue. Additionally, the incidence of inflammation-associated macrophage infiltration markers known as crown-like structures (CLS), which consist of dead adipocytes surrounded by macrophages (Bigornia et al. 2012), was evaluated (see, please, examples of IHC images in Fig. 1). Quantitation was made by two independent experts (M.M., L.M.B), which took into account intensity of staining and average percent of stained cells per unit of the section square.

In the course of preparation to real-time PCR for *UCP1*, *CYP19* (aromatase), *omentin* and *PTEN* genes *mRNA expression* evaluation a preliminary total RNA extraction from frozen omental fat tissue and complementary DNA (cDNA) analysis by reverse transcription reaction were performed. For this a 0.2 cm^3^ of fat were homogenized in 500 µl of TRIzol (Life Technologies), the lysate obtained was centrifuged at 12,000 rpm in +4 °C environment for 10 min, then the hypophase was transferred to clean test tube. To each tube 150 µl of 24:1 chloroform and isoamyl alcohol mixture was added. The sample was mixed and centrifuged at 12,000 rpm in +4 °C environment for 15 min and RNA-rich upper phase was collected. mRNA was precipitated by isopropyl alcohol in the presence of glycogen, rinsed by 70% ethanol and dissolved in water. Then, the RNA solution was diluted tenfold and used for complementary DNA (cDNA) synthesis in 20-µl reverse transcription reaction. The reaction mix included 10 µl of RNA solution, 10× buffer for reverse transcriptase (2.0 µl), M-MulV reverse transcriptase (Sibenzyme) (150,000 IU/ml) (1.0 µl), dNTP mix (10 mM of each nucleotide) (1.0 dNTP), random hexaprimers (conc. of 10 ODU/ml) (1.0 µl), and RNase inhibitor (5 IU/µl) (1.0 µl). The following temperature schedule of reverse transcription was used: 20 °C for 5 min, 38 °C for 30 min, 95 °C for 5 min. The cDNA sequences target and reference gene (SDHA) were amplified with specific primers in the presence of TaqMan probes (Table 1). Real-time PCR was performed with BioRad CFX96 Real-time PCR Detection System equipment in N.N.Petrov Research Institute of Oncology molecular oncology laboratory. A 20-µl reaction mix contained 1 µl of cDNA solution, 2.0 units of active Thermostar DNA polymerase, onefold PCR buffer, 2.5 mM of MgCl_2_, 200 µM of each NTPs, 300 nM of direct and reverse primers, and TaqMan probe. The following PCR amplification conditions were used: 20 s of denaturation at 95 °C, annealing and synthesis for 1 min at 60 °C, 45 cycles. The relative expression of each gene in samples was calculated by 2^−∆Ct^ formula (where Ct means Cycle threshold, ∆Ct = Ct (target gene)—Ct (reference gene, SDHA, succinate dehydrogenase catalytic subunit).

**Table 1:** Primer sequences used for studied genes mRNA expression assay

Primer	5′-3′ sequence	Fragment size in base pairs
UCP1-ex3/4_F	GTCTTTGGAAAGGGACTACT	125
UCP1-ex5/6_R	GGGGACGTCATCTGCTAATA	
UCP1-P	FAM-AGTGTCATCATCAATTGTACAGAGCT-BHQ	
CYP19A1 ex3/4-F	TTATCAGCAAGTCCTCAAGTAT	179
CYP19A1 ex4/5-R	GGGGCCTGACAGAGCTTTC	
CYP19A1 ex4-P	FAM-AGGCATCATATTTAACAACAATCCAGA-BHQ	
Omentin-F	AACAGCTCCCTGCTGAGGTA	199
Omentin-R	GCTGGCCATAGGGTGAGTAA	
Omentin-P	FAM-CATCTACCAGAAATATCCAGTGAAAT-BHQ	
PTEN ex 5/6-F	GAGACAAAAAGGGAGTAACTAT	155
PTEN ex 6/7-R	ATTGCAAGTTCCGCCACTGA	
PTEN ex6-P	FAM-TATAGCTACCTGTTAAAGAATCATCTG-BHQ	
SDHA-F	CCACTCGCTATTGCACACC	102
SDHA-R	CACTCCCCGTTCTCCATCA	
SDHA-P	JOE-ACGGTCTCTGCGATATGATACCA-BHQ	

*UCP1* uncoupling protein one, *CYP19A1* cytochrome 19, aromatase, *PTEN* phosphatase and tension homolog, *SDHA* succinate dehydrogenase catalytic subunit

During statistical analysis parametric (Student’s *t* test) and—in samples without normal distribution—nonparametric methods (mostly χ^2^ test, Pearson criterion and Fisher’s exact test with Yates correction) were used. Calculations were made with Statistica, v.10 and SigmaPlot software. The changes were considered statistically significant if *p* value was ≤0.05. The study of correlations between omental fat state and tumor characteristics relied on Spearman’s correlation coefficient calculations. All patients included in the study signed a consent form, although the specific informed consent obtainment was not mandatory since personal data of individual patients were not presented. An approval of Local Ethics Committee was obtained for this study in accordance to the principles formulated by the 1964 Helsinki declaration and its later amendments, which were strictly followed in the course of the investigation. All procedures performed in studies involving human participants were compatible with institutional and national research committees’ ethical standards.

## Results

In the course of these studies, several directions in omental fat evaluation were exploited, in particular: (a) comparison of this tissue characteristics in patients with different BMI values and signs of SO or MHO obesity; (b) comparison of results in patients with endometrioid and non-endometrioid endometrial carcinomas; (c) establishing by correlation analysis the connection between expression of studied omental fat markers and cancer characteristics (clinical stage and differentiation grade).

According to *immunohistochemistry* data analysis (Table 2) there were no distinctions in omental fat expression for any of the proteins studied (UCP1, aromatase, CD68 and CD163) between endometrioid and non-endometrioid uterine body cancer patients. Also, the same methodology did not yield evidence in favor of BMI increment correlation to changes in expression of aromatase/estrogen synthetase, markers of tissue macrophage infiltration (CD68, CD163) as well as UCP1 (although latter parameter demonstrated a higher mean value in EC patients with normal/<25.0/BMI, this observation most probably could be explained by prominent dispersion of individual values). However, when we switched from BMI values to obesity phenotypes, SO patients’ population demonstrated statistically significant increase in aromatase and macrophage marker CD68 expression compared to MHO group. In SO group, there was also a tendency to higher UCP1 protein expression (Table 2). This latter result has been somewhat unexpected since UCP1 protein is responsible for heat generation in brown adipose tissue (along with uncoupling of the respiratory chain) and has several other functions (Oberkofler et al. 1997; Cannon et al. 2006), see “Discussion” section.

**Table 2:** Expression of studied proteins in omental fat of endometrial cancer patients with different body mass index values and obesity phenotypes (*immunohistochemistry*)

Group	Expression score for proteins studied (M ± m)				BMI (M ± m)
	UCP1	Aromatase	CD68	CD163	
BMI <25.0 (8)	0.71 ± 0.41	0.81 ± 0.28	1.31 ± 0.28	2.00 ± 0.27	22.68 ± 0.46
BMI 25.0–29.9 (14)	0.46 ± 0.17	0.71 ± 0.16	0.79 ± 0.20	2.00 ± 0.21	28.63 ± 0.27
BMI ≥30.0 (28)	0.39 ± 0.10	0.88 ± 0.28	1.22 ± 0.19	2.43 ± 0.12	36.09 ± 1.05
SO (31)	0.48 ± 0.11^1^	0.93 ± 0.12^2^	1.26 ± 0.18^3^	2.39 ± 0.13	34.84 ± 1.10^4^
MHO (11)	0.23 ± 0.10^1^	0.50 ± 0.17^2^	0.60 ± 0.21^3^	2.00 ± 0.19	30.11 ± 0.80^4^
Endometrioid carcinomas (37)	0.49 ± 0.12	0.77 ± 0.11	1.00 ± 0.12	2.17 ± 0.12	32.45 ± 1.23
Non-endometrioid carcinomas (13)	0.41 ± 0.11	1.05 ± 0.22	1.27 ± 0.31	2.38 ± 0.21	30.00 ± 1.21

Notes: The immunohistochemistry staining evaluation was based on a score system (result/score): (−) 0, (±) 0.5, (+) 1, (++) 2, (+++) 3, (++++) 4; *BMI* body mass index, *SO* “standard” obesity, *MHO* “metabolically healthy” obesity. Aromatase—estrogen synthetase; UCP1—an “uncoupling” protein involved in thermogenesis; CD68—a marker mostly expressed by macrophages (predominantly M1 type), including macrophages infiltrating fat tissue; CD163–M2 type macrophages marker [see (Bigornia et al. 2012) and “Discussion” section]. *p* (1) < *0.1; p* (2, 3, 4) < 0.05. Number in brackets is the number of cases

Of note, in spite of above mentioned increase of CD68 expression in omental fat of SO group, the expression of other proinflammatory marker, crown-like structures (CLS), in this tissue was the same for both SO and MHO patients (CLS were found in 19.4 and 18.2% of cases, respectively). However, according to available data (Bigornia et al. 2012), CLS density of greater omentum fat tissue is much less pronounced compared to other visceral and subcutaneous fat tissue depots, and this probably makes the CD68 expression data obtained by us in patients with different obesity phenotypes (Table 2) more tenable.

In comparison to immunohistochemistry data analysis, the data on *studied genes mRNA expression* in omental fat tissue yielded only a tendency to increase in UCP1 and (less so) omentin signal intensity in EC patients with BMI values >25.0. No difference in mRNA expression data was found in omental fat in relation to tumor morphology (endometrioid vs non-endometrioid) and obesity type. Although arithmetic mean mRNA values of studied samples seemed to be different, it was just an effect of the values distribution which was far from normal (or Gaussian) that could easily be proved by Fischer’s exact test and χ^2^ test results (Table 3).

**Table 3:** mRNA expression of studied genes in omental adipose tissue samples from endometrial cancer patients with different body mass index and obesity phenotype (real-time PCR)

Group	mRNA expression value in units, 2^−∆Ct^ (M ± m)				BMI (M ± m)
	*UCP1*	*CYP19*	*Omentin*	*PTEN*	
BMI <25.0 (9)	0.005 ± 0.002^1^	0.005 ± 0.002	0.383 ± 0.152^2^	1.029 ± 0.620^IY^	22.99 ± 0.38
BMI 25.0–29.9 (16)	0.022 ± 0.008^1, I^	0.008 ± 0.002	1.268 ± 0.476	3.400 ± 1.540	28.54 ± 0.26
BMI ≥30.0 (32)	0.919 ± 0.900^I^	0.010 ± 0.003	0.752 ± 0.318	4.920 ± 3.372	36.41 ± 1.11
BMI ≥25.0 (48)			0.932 ± 0.265^2^	4.473 ± 2.269^IY^	
SO (33)	0.019 ± 0.006^II^	0.007 ± 0.001	0.843 ± 0.300	4.242 ± 3.220	34.62 ± 1.11
MHO (14)	1.642 ± 1.620^II^	0.015 ± 0.007	1.161 ± 0.564	4.629 ± 1.780	31.65 ± 1.54
Endometrioid carcinomas (41)	0.592 ± 0.579^III^	0.008 ± 0.003	0.802 ± 0.284	2.208 ± 0.807^Y^	32.76 ± 1.13
Non-endometrioid carcinomas (14)	0.030 ± 0.013^III^	0.010 ± 0.002	0.960 ± 0.394	7.569 ± 5.885^Y^	29.61 ± 1.62

A relative expression of each gene in samples was calculated using a 2^−∆Ct^ formula (where Ct is cycle threshold), ∆Ct = Ct (target gene)—Ct (reference gene, SDHA, see “Methods and patients” section). Each of subgroups lacked an expression in some samples; mean values are given for cases with gene expression

*BMI* body mass index, *SO* “standard” obesity, *MHO* “metabolically healthy” obesity. Numbers of cases are presented in brackets. UCP1—see notes to Table 2. *CYP19* cytochrome 19, aromatase; Omentin—adipokine (fat tissue hormone initially discovered in a great omentum depot, see “Discussion” section); *PTEN* phosphatase (Phosphatase and tensin homolog) gene, whose mutation or lowered expression quite often is found in malignant tumors, including endometrial cancer (see “Discussion” section)

Student’s *t*-test: *p* ^*1*^ *0.05; p* ^*2*^ *0.06.* Fischer’s exact test (by number of cases ≥ median for the whole group): ^I^ p 0.53; ^II^ p 0.50; ^III^ p 0.073 (in favor of non-endometrioid tumors due to one extremely different value in endometrioid carcinomas group); ^IY^ *p* 0.32 (Chi-square *p 0.39*); ^Y^ p 0.59

On the other hand, according to the *rank correlation analysis* UCP1 expression in omental fat was more pronounced in patients with advanced clinical stages of EC, and this was more evident in MHO group (Table 4). *Omentin* mRNA expression in omental fat depot demonstrated a modest tendency to positive correlation with EC stage in SO group, although fat tissue *omentin* mRNA level correlated to its serum values only in patients with MHO (correlation coefficient *ρ* = 0.44), but not in SO patients (*ρ* = 0.05). In addition, an inclination to correlation between expression levels in omental adipose tissue and better tumor differentiation (G1) was found for UCP1 and CD68 protein in patients with MHO and with worse (high grade, G3) differentiation—for *UCP1* mRNA (in MHO group) and CD68 protein expression (in SO group) (Table 4).

**Table 4:** Spearman rank correlation of omental fat markers expression with tumor characteristics in endometrial cancer patients with ‘standard’ (SO) or ‘metabolically healthy’ (MHO) obesity

Group	Immunohistochemistry, proteins				mRNA (correction by reference gene)			
	UCP1	Arom	CD68	CD163	UCP1	Arom (CYP19)	omentin	PTEN
SO								
Differentiation (G)	−0.006 (30)	0.063 (29)	0.277 (25)	0.158 (31)	−0.294 (21)	0.224 (24)	0.168 (30)	−0.174 (22)
Clinical stage	0.227	−0.113	−0.091	0.172	0.303	0.010	0.253	−0.170
MHO								
Differentiation (G)	−0.401 (11)	0.040 (10)	−0.262 (10)	0.026 (11)	0.365 (11)	−0.071 (11)	−0.179 (12)	0.000 (12)
Clinical stage	0.596	0.255	0.0392	−0.099	0.786	−0.170	0.111	−0.123

See the “Methods and patients” section and notes to the Tables 2 and 3 for data evaluation methods explanation

In brackets: number of studied patients (samples)

## Discussion

In endometrial cancer patients the omental fat depot can be considered from at least two points of view: as an important region for metastatic spread [even on relatively early cancer stages (Joo et al. 2015)] and as a source of hormone-like and pro-inflammatory regulatory signals able to influence endometrial carcinogenesis and tumor progression (Klopp et al. 2012). The second of these “characteristics” is as yet much less studied (Klopp et al. 2012; Zemlyak et al. 2012). However, considering the role of excessive fat mass as an EC risk factor (Bokhman 1983; Sherman 2000; Brinton et al. 2013; Setiawan et al. 2013; Fader et al. 2009) and the fact of obesity heterogeneity (Berstein 2012), the research of omental fat features in EC patients is undoubtedly important at least since this fat tissue depot is located nearby from the uterus.

Here we should mention several factors, which were also the stimulus for us to conduct this research. F.e., no studies of this kind had been made which took into account different phenotypes of obesity. Further, according to some data [in particular, a comparative data on omental and subcutaneous fat tissue *UCP1* expression (Esterbauer et al. 1998)], the greater omentum fat depot may have brown as well as white fat tissue properties. Multilocular adipocytes, which are characteristic for brown fat tissue, are found though in omental fat only in very special situations (Frontini et al. 2013). Nevertheless, the supposed plasticity or trans differentiation of adipocytes and their predecessors [allowing also white fat tissue cells acquire some brown fat cells characteristics (Smorlesi et al. 2012)] draws additional attention to omental fat functional abilities, which determine whether it is closer to white or brown fat tissue in postmenopausal EC patients. Therefore, this was another reason for interest in fat tissue markers (especially UCP1 and omentin) expression comparison in EC patients with “standard” and “metabolically healthy” obesity.

There are some notable and previously undescribed aspects of this research, which constitute its strengths. First, while omental fat tissue *immunohistochemistry* data analysis did not reveal connection between studied characteristics and body mass index or EC morphology, the fat tissue depot samples obtained from patients with MHO were characterized by lower—than in SO group—aromatase (estrogen synthetase) and pro-inflammatory macrophage marker CD68 expression. There was also a tendency to lower UCP1 expression in patients with MHO (Table 2).

The mRNA expression was also studied, although this data (on the basis its mean values) turned out to be less informative. It confirmed IHC data on the absence of distinctions in omental fat characteristics between patients with endometrioid and non-endometrioid carcinomas, while yielded no differences between SO and MHO groups (Table 3) in spite of just mentioned distinctions discovered by immunohistochemistry assay. In particular, mRNA data did not demonstrate in these groups any difference in expression of *aromatase* (estrogen synthetase) and *PTEN* genes (Table 2). The latter is a tumor suppressor gene subject to negative regulation by Akt/PKB signaling pathway (Tokunaga et al. 2008). Although PTEN is undoubtedly involved in EC carcinogenesis (Sherman 2000; Feng et al. 2012), there is only scarce data on its tumor expression in obese EC patients [see (Westin et al. 2015)] and no publications describing PTEN expression in adipose tissue of obese/non-obese patients with EC.

One of the conclusions from our results (showing in this case the greater practical value of immunohistochemistry compared to mRNA expression study, see Tables 2 and 3) is a necessity of studying omental fat samples in EC patients not only for *PTEN* gene, but also for its protein expression. The same conclusion can be made in regard of omentin IHC studies advisability, since—as had been discovered by us—a connection between this adipokine mRNA expression in omental fat and clinical-morphological EC features was rather modest (Table 4). Of note, in EC patients’ population we could not find any association between serum omentin concentration or its mRNA expression in omental tissue samples and BMI value (data not presented) in spite of published evidence of negative correlation between serum omentin level and excessive body mass (Tan et al. 2008) [although there is also a report stating the contrary (Choi et al. 2011)]. One also should not forget that while omentin is commonly viewed as a typical visceral adipokine, it (as already mentioned in Introduction) is mostly produced by fat tissue stromal cells but not adipocytes (Yang et al. 2006). Its serum values vary up and down in different cancer patients populations (Uyeturk et al. 2014; Shen et al. 2016), although so far no data on this subject was presented in regard of EC patients.

Also, as the analysis of immunohistochemistry and mRNA expression data in omental depot of patients with endometrioid and non-endometrioid cancer found no evident difference [indirectly confirming recent data which did not demonstrate significant distinctions in obesity prevalence between patients with EC type I or II (Setiawan et al. 2013)], it may be an additional argument to consider obesity phenotype as a deserving attention factor able to influence omental fat tissue characteristics in the capacity of potential endometrial carcinoma clinical course modifier. Indeed, we managed to find a positive correlation between UCP1 expression on protein as well as mRNA level in omental fat and endometrial cancer clinical stage. The correlation for UCP1 with clinical stage was stronger in “metabolically healthy” patients than in females with “standard” obesity (Table 4). Therefore, although “metabolically healthy” obesity phenotype supposedly could be characterized by higher brown fat contents (Berstein 2012), UCP1 in this case looks rather like a marker of more “problastomogenic” visceral fat tissue, for which the omentum serves as one of depots.

There are several arguments in favor of this suggestion. First, the co-cultivation of omental adipose stromal cells (ASC) with endometrial cancer cell line Hec1a stimulates cell proliferation in a greater degree than co-cultivation with subcutaneous fat ASCs (Klopp et al. 2012). In addition, the UCP1 hyperexpression by tumor-associated stromal fibroblasts may potentiate cancer growth by providing high-energy nutrients in a paracrine fashion (Sanchez-Alvarez et al. 2013). On the other hand, these are certainly not final and only arguments as, for example, there is also data on exactly opposed (antitumor like) influence exerted by UCP1 (Chen et al. 2009). Besides, the fact of positive correlation between UCP1 expression level in omental fat and EC clinical stage, which is more evident—as was just mentioned—in MHO patients, cannot abolish our earlier data, according to which endometrial cancer clinical stage is more advanced in patients with “standard” (insulin resistance-associated) obesity (Berstein et al. 2015). In addition to this, among other studied markers as rather logical seem opposite associations of macrophage lineage label CD68 with EC differentiation grade (low and high, respectively) in females with MHO and SO (Table 4).

In summary, the EC patients omental fat tissue depot characteristics are influenced by obesity phenotype of the patient and have a certain correlation with tumor features. These observations can be—among other mechanisms related to adipocytes themselves—explained, in particular, by effect of some mediating factors associated with macrophage infiltration (Tables 2, 4) or so-called adipose inflammation (Howe et al. 2013; Linkov et al. 2014). These assumptions need further investigation of omental fat in different obesity phenotypes, especially in context of potential preventive or therapeutic interventions. Presented data are not likely to be contested by certain limitations of this study (in particular, the already mentioned fact of certain omental fat tissue markers, e.g. omentin, being studied only on mRNA, but not protein expression level). An expanding signs of EC molecular diversity [see f.e. data on POLE gene mutations analysis (Cancer Genome Atlas Research Network et al. 2013; Murali et al. 2014)] is also ‘an item’ to be considered during analysis of obesity connection with risk factors, as well as with clinical and morphologic features of EC. This fast accumulating evidence attracts additional attention to functional state of white and brown fat tissue in patients with different (and growing in number) EC types with the aim to compose useful practical recommendations.
